# Potential toxicity of *Schisandra chinensis* to water environment: acute toxicity tests with water crustaceans

**DOI:** 10.1007/s11356-023-30182-8

**Published:** 2023-10-14

**Authors:** Jana Valíčková, Štěpán Zezulka, Eliška Maršálková, Josef Kotlík, Blahoslav Maršálek, Radka Opatřilová

**Affiliations:** 1 Institute of Chemistry and Technology of Environmental Protection, Faculty of Chemistry, Brno University of Technology, Purkyňova 464/118, 61200 Brno, Czech Republic; 2 Department of Experimental Phycology and Ecotoxicology, Institute of Botany, Czech Academy of Sciences, Lidická 25/27, 60200 Brno, Czech Republic; 3 Department of Chemical Drugs, Faculty of Pharmacy, Masaryk University, Palackého třída 1946/1, 61200 Brno, Czech Republic

**Keywords:** Adaptogen, Lignan, Schisandrin, Zooplankton, Acute toxicity

## Abstract

Fruits of *Schisandra* *chinensis*, an East Asian liana plant, are currently more and more used to produce nutrient supplements that positively affect human health due to the content of various secondary metabolites. On the other hand, these substances because of their bioactivity can cause possible allelopathic or toxic effects concerning other organisms (algae, plants, animals). But the ecotoxicological properties of *S. chinensis* outside its area of origin have yet to be sufficiently verified. Two crustaceans, *Daphnia magna* and *Thamnocephalus platyurus*, were selected as model aquatic organisms to test the potential impact of *S. chinensis* active compounds on the aquatic environment. Crude water extract from *S. chinensis* fruits, simulating the natural leakage of active substances in water, was tested in treatments from 0.0045 to 45 mg/L (according to the content of schisandrin as the dominating lignan). Effective concentration (EC_50_) causing 50% lethal effect for *D. magna* was established to 0.0448 mg/L after 24 h and 0.0152 mg/L after 48 h. EC_50_ for *T. platyurus* reached 0.4572 mg/L after 24 h, i.e. more than ten times higher than for *D. magna*. This study showed that the potential environmentally relevant concentrations of *S. chinensis* bioactive compounds could represent a severe risk to aquatic ecosystems.

## Introduction

*Schisandra chinensis* (known as magnolia vine or wuweizi) is a liana used as a natural herbal medicine to treat many human diseases. This plant grows wild and is cultivated in the Far East, like northeastern China, Japan and Korea (Panossian and Wikman 2008). A work by Li Shih-Chen, “Pên T'shao Kang Mu”, on Chinese medicine published in 1596, contains the first written mention of the *Schisandra* species (Szopa et al. 2017). However, the seeds of *Schisandra* plants were also discovered in Europe, as confirmed (Teodoridis 2005) in a morphological and anatomical study of the holotype of *S. moravica* from Šafov and additionally seeds of the same species from the Cheb, Sokolov and Most Basins (Czech Republic) where the assignment of the seeds to the genus *Schisandra* MICH was confirmed.

As confirmed by many studies, *S. chinensis* is a promising plant with a positive effect on human health and is classified as an adaptogen. Hancke et al. (1999) describe the proven positive impact of *S. chinensis* on human health and focus on anti-hepatotoxic, antioxidant and anti-tumour effects and on improving physical performance and effects on the central nervous system. Ma et al. (2023) have shown in the study that *S. chinensis* has promising clinical efficacy in treating diabetes mellitus as one of the most common diseases in our current civilisation. *S. chinensis* and its compounds affect the endocrine system, e.g. cortisol and testosterone levels, and the metabolism of lipids and muscles (Leis et al. 2020). Another advantage of *S. chinensis* and its derivatives is that it can be a source of natural antioxidants (Wang and Wang 2013).

Plants from *Schisandraceae* family contain many pharmacologically usable substances including schisandrin, deoxyschisandrin, gomisin A, gomisin O and gamma-schisandrin (Smejkal et al. 2010; Wang et al. 2018). Most of the bioactive compounds can be found in fruits and seeds, lower contents are in leaves and stem (Lee et al. 2022). The real content of schisandrin as the dominant compound and other lignans found in the fruits of magnolia vine is highly variable. It depends on many factors, such as the method and place of cultivation or the fruit ripening stage. According to the literature sources, the content of schisandrin can be from 2.1 mg/g (Kohda et al. 2012) to 5.0 mg/g of dry fruit weight (Slanina et al. 1997).

Growing demands for the production of food supplements containing bioactive substances from this plant promote its farming in larger areas outside its place of origin. It evokes a question if *S. chinensis* can influence the environment in such sites. Schisandrin and other active compounds from *S. chinensis* belong to lignans, secondary plant metabolites known as allelochemicals (Costas-Gil et al. 2018; Scavo et al. 2019a; Scavo et al. 2019b) which can be toxic not only to plants but even to other organisms in the ecosystem. Currently, the data about the allelopathic effect of schisandrin are scarce.

*S. chinensis* grows wild in East Asia, but in some areas, it is farmed. For example, the average size of the farmland for *S. chinensi*s cultivation in South Korea was 0.5 ha per farm (Choi et al. 2015) and the scale of cultivation areas is still rising. In European area, *S. chinensis* is often planted only as an individual plant nearby garden ponds but because of the increasing inquiry on the fruits, it is considered its farming on larger areas. However, any study does not address the possible impact of *S. chinensis* farming on the surrounding environment.

Delicate water ecosystems can be affected by the effects of lignans leaking from fruits accidentally falling into the water body as proved on *Lemna minor* (Valickova et al. 2023). The effects of bioactive substances from *Schisandra chinensis* on aquatic animals are insufficiently researched and this lack of ecotoxicological data should be addressed.

An integral part of aquatic ecosystems and food webs is also invertebrate zooplankton species like *Daphnia magna* and *Thamnocephalus platyurus.* Because of their high sensitivity to environmental pollution, these species are included in standardised acute toxicity tests (Kerberova et al. 2022; Szklarek et al. 2022).

This study targets to assess the possible acute toxicity of aqueous extract from *S. chinensis* fruits to indicator zooplankton species *Daphnia magna* and *Thamnocephalus platyurus* to enhance the knowledge about the possible ecotoxicological risks of *S. chinensis* farming outside its areas of origin.

## Materials and methods

### Plant material and extraction

The ripe fruits of *S. chinensis* were harvested from a plant grown in the area of Vracov, Czech Republic. The whole fruits (including seeds) were freed from impurities, detached from the stalks and left to dry naturally in the air. The average portion of their dry mass was established at 20% of the fresh weight.

Crude aqueous extract from *S. chinensis* fruits (SCE) was obtained by a Soxhlet extraction with distilled water as described in Valickova et al. (2023). Its composition was analysed using high-performance liquid chromatography. HPLC (Agilent 1100 Series, Agilent Technologies, USA) was equipped with C18 column (Kinetex® 2.6 μm C18 100Å, LC Column 150 × 2.1 mm). Mobile phases were MilliQ water and methanol (70 to 100 %). Diode array detector was set to 225 nm. Calibration was done using external standard (CAS 7432-28-2, purity ≥ 98%, Merck). The content of the dominating compound schisandrin was determined to be 45 mg/L.

### Acute toxicity tests

Daphtoxkit F® and Thamnotoxkit F® (Microbiotests, Belgium) were used to test the acute toxicity of SCE to freshwater crustaceans *Daphnia magna* and *Thamnocephalus platyurus*. According to producer’s guides, resting stages of both organisms (*D. magna* ephippia and *T. platyurus* cysts included in the kits) were re-activated in a standard freshwater and cultivated on Petri dishes under continuous light for obtaining a population of adult animals (*D. magna* for 72 h, *T. platyurus* for 20–22 h).

The SCE was diluted with standard freshwater to the concentrations 0.0045 mg/L, 0.009 mg/L, 0.045 mg/L, 0.09 mg/L, 0.45 mg/L, 0.9 mg/L, 4.5 mg/L and 9 mg/L. Undiluted SCE (45 mg/L) was included in both tests too. Tested solutions and animals were transferred to test plates (4 wells per each treatment with 5 individuals per well for *D. magna*; 3 wells with 10 individuals per well for *T. platyurus*) and the cultivation continued in the darkness and controlled temperature (*D. magna* 20 ± 2 °C, *T. platyurus* 25 °C). According to producer’s guides and ISO standards (ISO 6341: 2012; ISO 14380: 2011), the mobility of *D. magna* was evaluated after 24 and 48 h and the mortality of *T. platyurus* was assessed after 24 h. The tests were run in triplicates.

### Statistics

The effect of SCE on both species was evaluated as the percentage of immobilised/dead individuals in each treatment according to the following equation:

$$Inhibition\left(\%\right)=\left(1-\left(N_L/N_T\right)\right)\times100\%$$

where *N*_L_ is the number of living individuals and *N*_T_ is the number of total individuals in each treatment.

The software STATISTICA (StatSoft Inc.®) was used to evaluate the obtained results statistically. The significance of the differences in the average values between the treatments was assessed by the non-parametric Kruskal-Wallis test and multiple comparison method (*P* < 0.05). The effective concentrations EC_50_/LC_50_ for both species were evaluated using GraphPad Prism® software (Dotmatics) by a dose-response curve and non-linear regression analysis.

## Results

### *Acute toxicity to Daphnia magna*

**Fig. 1:**
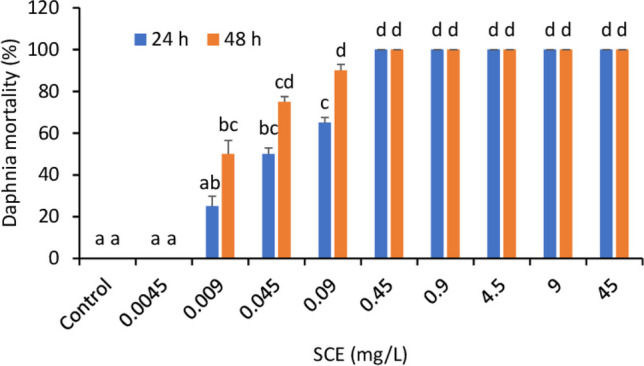
Acute toxicity test of SCE on *Daphnia magna* after 24 and 48 h. Data points represent the mean over twelve replicates, and standard deviations are indicated by error bars. Different letters mark significant differences between treatments within the time point (Kruskal-Wallis test, *P* < 0.05)

In accordance with the standards ISO 6341: 2012 and OECD Guidelines Test No. 202, daphnids were exposed to SCE in an acute toxicity test. The number of dead and immobilised individuals was assessed after 24 and 48 h (Fig. 1). The lowest tested treatment (0.0045 mg/L SCE) did not influence the vitality of daphnids either after 24 h or 48 h as compared to the untreated control. The number of surviving individuals decreased in 0.009 mg/L SCE treatment after 24 h by 25% and after 48 h by 50%. Higher treatments of 0.045 and 0.09 mg/L SCE caused more than 50% mortality on both days. From 0.45 mg/L SCE, all treatments exhibited 100% mortality. Undiluted SCE 45 mg/L caused even the destruction of the daphnid’s bodies.

The effective concentration (EC_50_) for *Daphnia magna* was established at 0.0448 mg/L after 24 h and 0.0152 mg/L after 48 h (Table 1), showing high acute toxicity of SCE to this invertebrate.

**Table 1:** Evaluation of SCE acute toxicity in tests with freshwater crustaceans

Organism	EC_50_/LC_50_ (mg/L)	Std. error	*R*^2^	Confidential intervals
*D. magna* (24 h)	0.0448	0.0022	0.9313	0.032–0.062
*D. magna* (48 h)	0.0152	0.0008	0.8921	0.011–0.023
*T. platyurus* (24 h)	0.4572	0.0274	0.9849	0.319–0.594

### *Acute toxicity to Thamnocephalus platyurus*

**Fig. 2:**
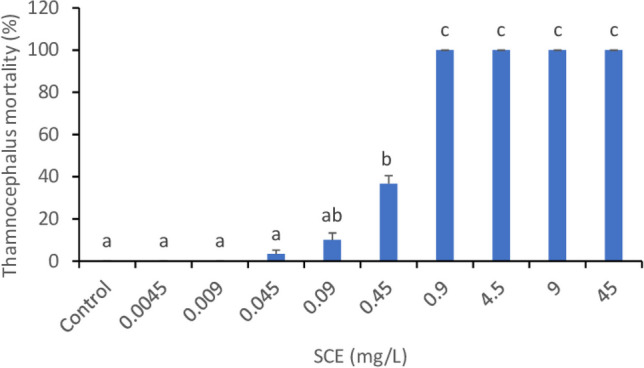
Acute toxicity test of SCE on *Thamnocephalus platyurus* after 24 h. Data points represent the mean over nine replicates, and standard deviations are indicated by error bars. Different letters mark significant differences between treatments (Kruskal-Wallis test, *P* < 0.05)

Following the ISO standard 14380: 2011, *T. platyurus* larvae were exposed to SCE in an acute 24 h toxicity test (Fig. 2). The lowest tested treatments (0.0045 and 0.009 mg/L SCE) did not influence the vitality of *T. platyurus* larvae after 24 h as compared to untreated control. Treatments 0.045, 0.09 and 0.45 mg/L SCE caused a decrease of surviving individuals’ number by 3, 10 and 36%, respectively. In higher treatments (0.9 to 45 mg/L SCE), the mortality reached 100%.

The effective concentration (EC_50_) for *T. platyurus* was established at 0.4572 mg/L after 24 h (Table 1).

## Discussion

Biologically active substances contained in plants, fruits or seeds represent a wide range of chemicals which can be helpful for humans but toxic for other organisms in the environment. One group of these secondary metabolites is lignans, compounds with allelopathic potential. Schisandrin and other lignans in *S. chinensis* fruits are highly beneficial for human health. Still, they can represent a severe risk to other organisms, as already proved for the aquatic plant *Lemna minor* (Valickova et al. 2023). Currently, the ecotoxicological data on the effects of plant lignans in a water environment on other organisms, including zooplankton, are scarce, and schisandrin still needs to be evaluated. But as Mayorga et al. (2010) and others stated, using complex whole plant extracts in ecotoxicological studies can be more suitable and significant than studying fractions because the individual constituents can lose their specific bioactivity when isolated from the crude extract.

Crustaceans represent an essential part of the zooplankton. *D. magna* and *T. platyurus* belong to the common species known for their sensitivity to pollution in the aquatic environment and are, therefore, often used as model organisms (Alvarenga et al. 2016; Szklarek et al. 2022). Therefore, this paper aimed to simulate the conditions when the extraction of compounds contained in *S. chinensis* fruits occurs accidentally in a water body and zooplankton species get in contact with it.

Both tested species exhibited considerable sensitivity when exposed to *S. chinensis* fruit extract. As shown in Table 1, after 24 h, the EC_50_ value for *D. magna* (0.0448 mg/L) was ten times lower than for *T. platyurus* (0.4572 mg/L), and after 48 h, the standard test period for *D. magna*, the EC_50_ value decreased even to 0.0152 mg/L. The exact mechanism of SCE toxicity to the crustaceans is currently unknown.

According to the literature sources (Slanina et al. 1997; Kohda et al. 2012), the content of schisandrin in *S. chinensis* fruits can reach 0.2 to 0.5% of their dry weight, i.e. one *S. chinensis* berry (average dry weight approx. 30 mg) can contain 0.06 to 0.15 mg of schisandrin. Hypothetically, when considering this schisandrin content, for reaching the schisandrin concentration in a water body close to the EC_50_ value for *D. magna*, only dozens of *Schisandra* berries could be sufficient to contaminate hundreds of litres of water.

The susceptibility of these species to various chemicals, including natural compounds, is different. In this case, *T. platyurus* seems to be less sensitive to SCE as compared to *D. magna,* similar to the results published on other organic pollution, like olive oil mill wastewater (Paixao et al. 1999), various organic matters like compost, digestate or sludge eluents (Alvarenga et al. 2016) or urban wastewater contaminated by artificial sweeteners (Kerberova et al. 2022). Opposingly, in the case of inorganic de-icing salts, *D. magna* was less sensitive than *T. platyurus* (Szklarek et al. 2022). Some examples of their differences in sensitivity to other contaminants are given in Table 2.

**Table 2:** Acute toxicity (EC_50_/LC_50_) of selected contaminants to freshwater crustaceans *D. magna* and *T. platyurus.* EC_50_ values are expressed as means

Contaminant		EC_50_ (mg/L; 48 h)	LC_50_ (mg/L; 24 h)	Reference
		*D. magna*	*T. platyurus*	
Tebuconazole	Fungicide	2.37	0.115	Tofan et al. (2023)
Nicotine	Alkaloid	0.789	Insensitive	Oropesa et al. (2017)
Amidine polystyrene	Nanoplastics	36.2	194.8	Saavedra et al. (2019)
Carboxyl polystyrene		111.4	318.2	
Atrazine	Pesticide	35.5	36.7	Palma et al. (2008)
Endosulfan sulphate		0.92	0.58	
Chlorpyrifos		0.74 × 10^−3^	0.53 × 10^−3^	

The effective concentration (EC_50_) of *Trapa japonica* leaves extract to *D. magna* was from 4 to 22 g wet mass per litre (Ishimota et al. 2019), and in contrast to this, EC_50_ for SCE to *D. magna* is 0.0152 mg/L (Table 1) corresponding to 15 to 37.5 mg fresh weigh per litre of *S. chinensis* berries. Similarly, the extract from *Hedychium coronarium* rhizomes exhibited a 50% mortality rate to *Daphnia similis* in concentration approx. 1.3 g fresh weight per litre (Costa et al. 2021). Both these results show that *S. chinensis* fruit extract is more toxic for daphnids than extracts from other plants.

In the case of *T. platyurus*, Mayorga et al. (2010) stated higher sensitivity of this species as compared to another crustacean *Artemia salina* when exposed to an extract of different Guatemalan plants. The lethal concentration (LC_50_) range was from 10 to 500 mg/L. Cangiano et al. (2002) described the toxic effect of diterpenes from *Ruppia maritima* and *Potamogeton natans* on green algae and a few zooplankton species, including *D. magna* and *T. platyurus.* In this case, *T. platyurus* was more sensitive than *D. magna,* and the LC_50_ value for the most toxic compound reached 0.84 μM, a lower value than in SCE 0.4572 mg/L (corresponding to 1 μM schisandrin).

Ji et al. (2014) described that only the minority of *S. chinensis* bioactive compounds (unmetabolised forms) were excreted via bile, urine and faeces in rats fed with *S. chinensis* extract. Similarly, Kim et al. (2014) stated only negligible excretion of *Schisandra* lignans via urine after oral and intravenous administration of *S. chinensis* extracts to rats. It can be hypothesised that the waste water contaminated by schisandrin used by humans as food supplements would be a negligible source of contamination, and the primary source of contamination would be *S. chinensis* farming in large areas due to fruit or other plant parts fallout.

## Conclusion

Although *S. chinensis* provides many beneficial effects for human health, it can harm aquatic ecosystems. Crustaceans *D. magna* and *T. platyurus* exhibited sensitivity to water-extractable bioactive compounds from *S. chinensis* fruits already in potentially environmentally relevant concentration. Reaction of other zooplankton species to SCE is unpredictable; nevertheless, the accidental contamination of water by *S. chinensis* bioactive compounds can disturb fragile food webs.

## Data Availability

Data will be made available on a reasonable request.
